# Comparison of prognosis between patients undergoing radical nephrectomy versus partial nephrectomy for renal cell carcinoma ≤7 cm T3aN0/xM0: Survival benefit is biased toward partial nephrectomy

**DOI:** 10.1002/cam4.4412

**Published:** 2021-11-14

**Authors:** Shiliang Liu, Chunxiang Feng, Chang Liu, Zhixian Wang

**Affiliations:** ^1^ Department of Urology Tongji Hospital Tongji Medical College Huazhong University of Science and Technology Wuhan China; ^2^ Department of Pathogenic Biology School of Basic Medicine Tongji Medical College Huazhong University of Science and Technology Wuhan China; ^3^ Department of Urology Union Hospital Tongji Medical College Huazhong University of Science and Technology Wuhan China; ^4^ Department of Geriatrics Tongji Hospital Tongji Medical College Huazhong University of Science and Technology Wuhan China

**Keywords:** kidney malignant, prognosis, SEER, surgery, T3a

## Abstract

**Background:**

There is limited and controversial evidence on the prognosis of partial nephrectomy (PN) versus radical nephrectomy (RN) in patients with T3aN0/xM0 renal cell carcinoma (RCC) upstaged from clinical T1 RCC. In this study, we aimed to assess the prognosis difference following PN versus RN in patients with ≤7 cm T3aN0/xM0 RCC.

**Methods:**

From the Surveillance, Epidemiology, and End Results database, a total of 3196 patients receiving treatment of PN/RN for ≤7 cm T3aN0/xM0 RCC with only extrarenal fat extension in 2010–2017 were identified. An inverse probability of treatment weighting (IPTW)‐adjusted cause‐specific Cox model with hazard ratio (HR) and 95% confidence interval (CI) was used for overall survival (OS) and cancer‐specific survival (CSS) analyses. Sensitivity analysis was based on the propensity score matching of PN and RN groups and from the dataset of 2010–2013.

**Results:**

A total of 872 patients underwent PN, compared with 2324 undergoing RN. After IPTW adjustment, there was no significant difference in preoperative baseline characteristics between the PN and RN cohorts. Patients who underwent RN had worse OS (HR_IPTW‐adjusted_, 1.46; 95% CI, 1.16–1.84; *p* = 0.001) and comparable CSS (HR_IPTW‐adjusted_, 1.03; 95% CI, 0.64–1.66; *p* = 0.890) than those receiving PN in all cohorts and subgroups with T3a RCC of ≤4 cm and perinephric fat extension. Further, in patients with 4–7 cm T3a RCC with perinephric‐fat invasion and all sizes of T3a RCC with sinus/perisinus fat extension, PN led to comparable OS and CSS. Sensitivity analyses validated these results.

**Conclusion:**

PN provides comparable CSS and OS or even better OS than RN for patients with RCC ≤7 cm T3aN0/xM0. Although our study has some limitations, our results indicated that PN might oncologically safe for clinical T1 RCC, even confirmed a pathologically T3a upstaging post‐PN.

## Graphical abstract

In clinical practice, it is not uncommon that clinical T1 renal cell carcinoma (RCC) has undergone pathologically upgrades to T3a after partial nephrectomy (PN) due to the limited ability of current imaging modalities to accuracy to predict the T3a stage preoperative. This fact has led to a treatment strategy dilemma: choice of clinical observation (keep PN unchanged) versus conversion to radical nephrectomy (RN) for such pathologic upstaging post‐PN. Whether a transformation of RN could provide an excellent prognosis for patients with clinical T1 RCC upstages to pathological T3a RCC is still to be warranted. In our study, we sought to investigate the prognosis of PN versus RN in ≤7 cm T3aN0/xM0 RCC. And we found that PN provided a comparable and even better overall survival than RN for these patients.

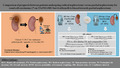

## 1 INTRODUCTION

Partial nephrectomy (PN) is the preferred treatment for T1 renal cell carcinoma (RCC).^1^ Although three‐dimensional reconstruction and virtual imaging techniques were reported to allow to facilitate the surgical planning preoperatively in complex renal masses evaluation,^2^, ^3^ there is still limited ability to accurately predict T3a stage disease preoperatively based on the current imaging modalities, it is not uncommon for T1 RCC to be upgraded to pathological stage T3a following PN, with reported incidence rates of 1.9%–14%.^4^, ^5^, ^6^, ^7^, ^8^, ^9^ This fact has led to a treatment strategy dilemma in clinical practice regarding whether it is preferable to keep PN unchanged, or change to radical nephrectomy (RN), since there is limited and controversial evidence of the surgical benefit of PN compared with RN for T3a RCC.^5^, ^8^, ^10^, ^11^, ^12^, ^13^, ^14^

Currently, there have been raised concerns about the oncological safety of PN for patients with T3a RCC.^5^, ^8^ In clinical practice, a transformation of surgery to RN from PN may not result in an excellent prognosis for patients with pathological T3a RCC upstaged from clinical T1 disease. Some prior studies have suggested that PN can yield a satisfactory prognosis for selected T3a RCC,^10^, ^11^, ^12^ patients who underwent PN in comparison to RN experienced similar or even better oncological outcomes in pathological T3a RCC upstaged from a clinical T1 disease.^8^ While some other studies have observed that RN is associated with a lower risk of recurrence than PN when conducted for clinical T1 tumors pathologically upgraded to T3a.^14^ Notably, small sample sizes and with a short follow‐up and had a selective bias resulting in confounding were the primary limitation for these studies. In addition, the influence of tumor diameter or extrarenal fat extension pattern on survival benefit from PN versus RN should be considered, since tumor diameter or extrarenal fat extension pattern have been identified as significant risk factors for survival outcomes in patients with T3a RCC.^15^, ^16^, ^17^, ^18^, ^19^, ^20^, ^21^, ^22^

In our present study based on a large sample size and a long follow‐up time, we want to furtherly investigate whether PN compared with RN can provide a comparable prognosis for patients with T3a RCC and to investigate if T3a tumor diameter and extrarenal fat extension pattern could impact the survival outcomes between T3a RCC patients treated with PN and RN. Here, to address these important questions, we used the Surveillance, Epidemiology, and End Results (SEER) database to evaluate differences in prognosis between patients with T3a RCC undergoing PN versus RN, and to investigate the influence of tumor diameter and extrarenal fat extension type on the survival benefit from PN versus RN for T3a RCC patients.

## 2 METHODS

### 2.1 Database and patient selection

The SEER‐18 registries database was screened to identify cases of pathological diagnosis of primary T3aN0/xM0 RCC between 2010 and 2017; case lists were identified from SEER using *Stat software (version 8.3.9). This study was approved by the Ethics Review Board of Tongji Hospital of Huazhong University of Science and Technology based on the Declaration of Helsinki.

**FIGURE 1:**
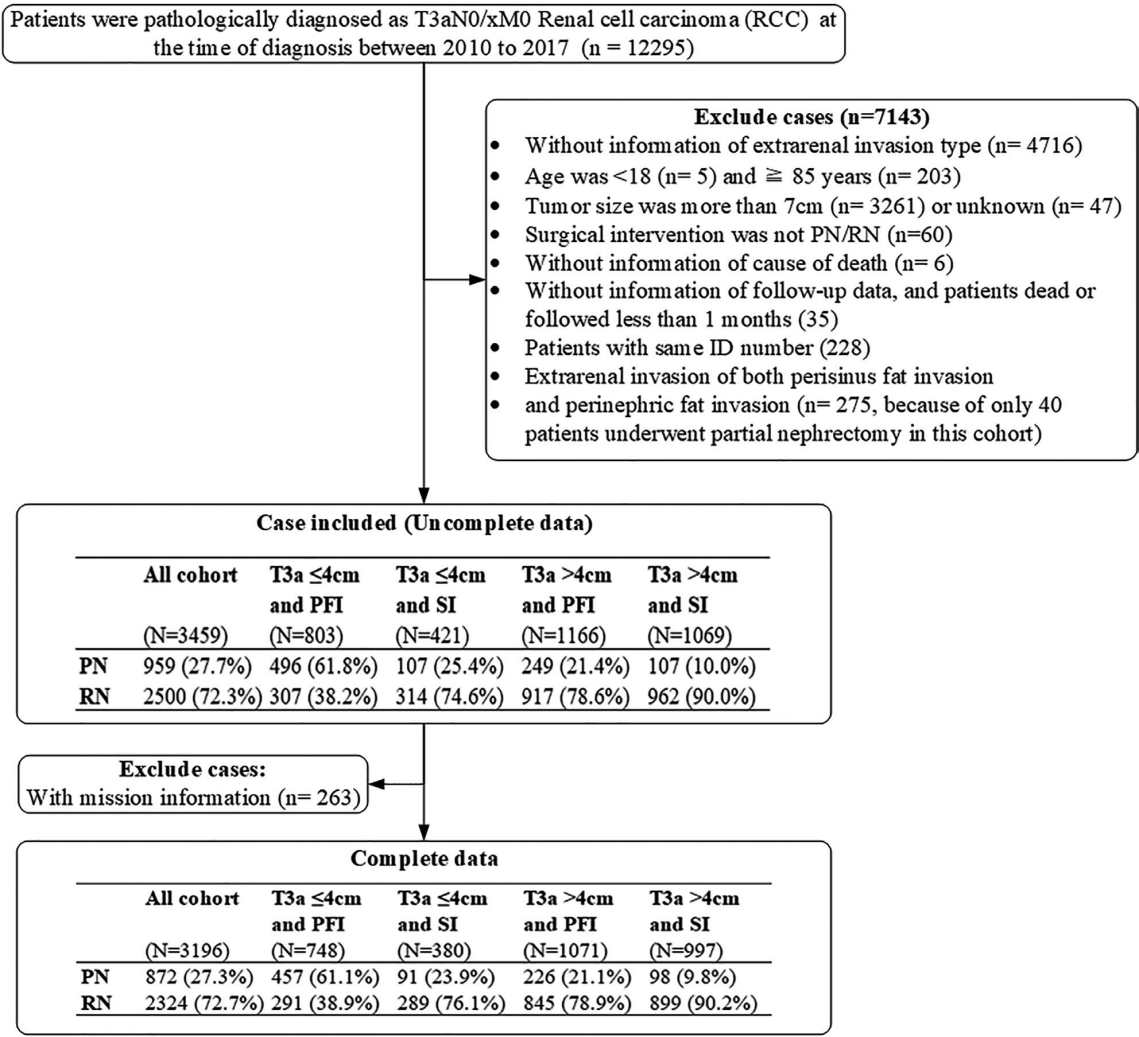
Flow chart showing the data screening and analysis workflow. FI, perinephric‐fat invasion; PN, partial nephrectomy; RN, radical nephrectomy; SI, sinus/perisinus invasion

The data selected from the SEER database are presented in Figure 1. Briefly, all RCC cases included in the study were pathologically confirmed T3aN0/xM0 RCC of ≤7 cm diameter, which is equivalent to clinical T1 RCC. All patients included for analysis in our study were aged 18–85 years and underwent PN or RN surgery. Patients were excluded if: (1) no information on extrarenal fat extension pattern was recorded; (2) the specific cause of death and follow‐up time were not recorded, or they were followed for <1 month after surgery or died within 30 days; or (3) the same patient identification numbers had multiple records in the case list.

### 2.2 Survival outcome

In our present study, the two primary survival outcomes of interest were overall survival (OS) and cancer‐specific survival (CSS). The *Cause of Death Recode* in the SEER was used to identify the cause of death. CSS was calculated according to “RCC‐cause mortality,” which was defined as patients who died from RCC, while patients who died from other causes were considered as “competing events” for CSS, and considered in the competing risk analysis. “All‐cause mortality” included patients who died from any cause of death, and were included for OS analysis. “Time of survival” was calculated from the diagnosis date to the date of end event occurrence or censor.

### 2.3 Analysis variables

The following demographic characteristics were collected: year at diagnosis (2010–2013 vs. 2014–2017); age at diagnosis (18–60 vs. 61–85 years); sex; race (White race vs. Black race vs. Other race), prior history of other system cancer (Yes vs. No) and median household income (<$59,999 vs. ≥$60,000).

Renal cell carcinoma‐related characteristics were included, as follows: tumor diameter (≤4 vs. 4.1–7 cm), tumor laterality (Right vs. Left), and RCC histology type (Clear cell carcinoma, Papillary carcinoma, Chromophobe carcinoma, and others); Fuhrman grade (I/II vs. GIII/IV); regional lymph nodes surgery (Yes vs. No); and extrarenal fat extension (sinus/perisinus extension and perinephric fat extension).

### 2.4 Statistical analyses

The continuous variables are described as mean (standard deviation) and were compared using a *t*‐test. If the data meets the normal distribution; while it is expressed as median (interquartile range [IQR]) and were compared with the Kruskal–Wallis test if it did not meet the normal distribution. A Chi‐square test was used for categorical variables comparison and is presented as frequencies (%).

Since this was not a randomized control study, treatment‐selection bias could have impacted the analysis of the difference in prognosis between patients undergoing PN versus RN. The inverse probability of treatment weighting (IPTW) method was used to control for such bias and to balance preoperative confounding factors between the PN and RN groups. First, a propensity score (PS) was calculated for each individual using multivariate logistic regression analysis based on the probability of surgical treatment by PN compared with RN; the preoperative covariables included in the model were: year, age, gender, race, median household income, prior history of other system cancer, tumor diameter, and tumor laterality. Although Fuhrman grade, RCC histology, and sarcomatoid dedifferentiation were identified postoperatively, they may still have impacted the surgical intervention in the clinical practice; therefore, these covariables were also included in the analysis model. Then, weights were calculated as 1/PS for PN individuals and 1/(1 – PS) for RN individuals. Finally, the IPTW approach was used to balance the difference in perioperative confounding factors between the PN and RN cohorts.

The reverse Kaplan–Meier method was used to evaluate the median follow‐up time. OS and CSS of the PN and RN cohorts were compared based on the IPTW‐adjusted Kaplan–Meier method with the log‐rank test. For the IPTW population, univariate and multivariable Cox proportional hazards regression models and cause‐specific Cox regression models were constructed to evaluate risk factors for OS and CSS, respectively, and hazard ratio (HR) with 95% confidence interval (CI) values calculated to compare the influence of PN versus RN on OS and CSS. For the non‐IPTW population, Fine and Gray competing risk regression models with a corresponding sub‐distribution hazard ratio were fitted to assess the risk of “RCC‐specific mortality” and “other causes of mortality”. Since tumor diameter and extrarenal fat extension pattern were significant predictors of surgery intervention, all analyses included all cohorts, with four subgroups: ≤4 cm T3a of perinephric fat extension, 4–7 cm T3a of perinephric fat extension, ≤4 cm T3a of sinus/perisinus extension, and 4–7 cm T3a of sinus/perisinus extension.

Furthermore, to maximize the accuracy of our hypothesis and ensure consistent results, we refitted the Cox and Fine and Gray models in two sensitivity analyses: (1) a model including unweighted data from 2010 to 2013, in which most patients were followed for >5 years; and (2) 1:1 propensity‐score matched Cox proportional hazards model and Fine and Gray competing risk regression models to evaluate the influence of PN versus RN on OS and CSS. For the propensity‐score matched analysis, year at diagnosis, age at diagnosis, gender, race, median household income, prior history of other system cancer, tumor diameter and tumor laterality, pathological T3a invasion type, Fuhrman grade, RCC histology, and sarcomatoid dedifferentiation were matched between PN and RN group. In addition, another 1:1 propensity‐score matching analysis between PN and RN did not include the covariables of postoperative (pathological T3a invasion type, Fuhrman grade, RCC histology, and sarcomatoid dedifferentiation).

R v.4.1.0 (www.r‐project.org) was used for Statistical Computing. All *p* values are two‐sided, and *p* < 0.05 was defined as statistically significant.

## 3 RESULTS

### 3.1 Baseline characteristics and treatment comparison

Table 1 showed the clinicopathologic characteristics of the total cohort (*N* = 3196). The median age at diagnosis was 65.0 (IQR, 57.0–73.0) years, and 65.5% of patients were 61–85 years. The male:female ratio was 2.21:1 (2200:996). Approximately 19.6% of patients had a history of other cancer. The median tumor diameter was 4.8 (IQR, 3.5–6.0) cm. Of T3a RCC 43.1% and 56.0% had sinus/perisinus extension and perinephric fat extension characteristics, respectively. In addition, 33.5%, 31.2%, 23.4%, and 11.9% of T3a RCC were 4–7 cm T3a of perinephric fat extension, 4–7 cm T3a of sinus/perisinus extension, ≤4 cm T3a of perinephric fat extension, and ≤4 cm T3a of sinus/perisinus extension, respectively. Further, regional lymph node removal was more common in patients with surgical treatment of RN than in those receiving PN surgery.

**TABLE 1:** Baseline characteristics, outcomes summary, and analysis of predictors for RN (vs. PN)

	All cohort	Subgroup by surgery types			Predictors for RN propensity vs. PN performed			
		PN	RN	*p* value	OR (95% CI)	*p* value	Adjusted OR (95% CI)	*p* value
*N*	3196	872 (27.3%)	2324 (72.7%)					
Prognostic information								
Median time to event/censor, median [IQR], years	3.50 [1.92;5.67]	3.83 [2.00;5.67]	3.42 [1.92;5.60]	0.034				
Estimated median follow‐up time, years	4.25	4.33	4.17	0.18				
Survival outcomes, *N* (%)				<0.001				
Alive	2583 (80.8)	760 (87.2)	1823 (78.4)					
Dead from RCC	243 (7.60)	34 (3.90)	209 (8.99)					
Dead from other events	370 (11.6)	78 (8.94)	292 (12.6)					
Clinicopathologic characteristics								
Year at diagnosis, *N* (%)				0.655				
2010–2013	1382 (43.2)	371 (42.5)	1011 (43.5)		1 reference		1 reference	
2014–2017	1814 (56.8)	501 (57.5)	1313 (56.5)		0.96 [0.82–1.13]	0.63	0.74 [0.62–0.89]	0.001
Age at diagnosis, median [IQR]	65.0 [57.0;73.0]	64.0 [56.0;71.0]	66.0 [57.0;73.0]	<0.001	1.02 [1.01–1.02]	<0.001	1.02 [1.01–1.03]^a^	<0.001
Age at diagnosis (category), *N* (%)				0.003				
18–60 year	1104 (34.5)	337 (38.6)	767 (33.0)		1 reference		1 reference	
61–85 year	2092 (65.5)	535 (61.4)	1557 (67.0)		1.28 [1.09–1.50]	<0.001	1.39 [1.16–1.68]	<0.001
Race, *N* (%)				0.394				
White	2726 (85.3)	734 (84.2)	1992 (85.7)		1 reference			
Black	245 (7.67)	68 (7.80)	177 (7.62)		0.96 [0.72–1.28]	0.78		
Other	225 (7.04)	70 (8.03)	155 (6.67)		0.82 [0.61–1.10]	0.18		
Sex, *N* (%)				0.003				
Female	996 (31.2)	236 (27.1)	760 (32.7)		1 reference		1 reference	
Male	2200 (68.8)	636 (72.9)	1564 (67.3)		0.76 [0.64–0.91]	<0.001	0.79 [0.65–0.95]	0.015
Median household income, *N* (%)				<0.001				
Less than $59,999	1205 (37.7)	277 (31.8)	928 (39.9)		1 reference		1 reference	
More than $60,000	1991 (62.3)	595 (68.2)	1396 (60.1)		0.70 [0.59–0.83]	<0.001	0.69 [0.58–0.83]	<0.001
With prior other cancer, *N* (%)				0.012				
No	2571 (80.4)	676 (77.5)	1895 (81.5)		1 reference		1 reference	
Yes	625 (19.6)	196 (22.5)	429 (18.5)		0.78 [0.65–0.94]	0.01	0.73 [0.59–0.91]	0.006
Laterality, *N* (%)				0.798				
Left	1619 (50.7)	438 (50.2)	1181 (50.8)		1 reference			
Right	1577 (49.3)	434 (49.8)	1143 (49.2)		0.98 [0.84–1.14]	0.77		
Size, median [IQR]	4.80 [3.50;6.00]	3.50 [2.60;4.50]	5.20 [4.10;6.10]	<0.001	1.92 [1.81–2.04]	<0.001	1.85 [1.73–1.97]^b^	<0.001
Size (category), *N* (%)				<0.001				
<4 cm	1128 (35.3)	548 (62.8)	580 (25.0)		1 reference		1 reference	
4–7 cm	2068 (64.7)	324 (37.2)	1744 (75.0)		5.09 [4.31–6.01]	<0.001	4.50 [3.77–5.37]	<0.001
Histological type, *N* (%)				<0.001				
Clear cell RCC	2214 (69.3)	523 (60.0)	1691 (72.8)		1 reference		1 reference	
Other/undefined	982 (30.7)	349 (40.0)	633 (27.2)		0.56 [0.48–0.66]	<0.001	0.72 [0.59–0.86]	<0.001
Pathological T3a invasion type, *N* (%)				<0.001				
Perinephric fat invasion	1819 (56.9)	683 (78.3)	1136 (48.9)		1 reference		1 reference	
Sinus/perisinus fat invasion	1377 (43.1)	189 (21.7)	1188 (51.1)		3.78 [3.16–4.53]	<0.001	3.45 [2.83–4.19]	<0.001
Group, *N* (%)				<0.001				
≤4 cm T3a of FI	748 (23.4)	457 (52.4)	291 (12.5)		1 reference		1 reference	
≤4 cm T3a of SI	380 (11.9)	91 (10.4)	289 (12.4)		4.99 [3.78–6.58]	<0.001	4.90 [3.69–6.52]^c^	<0.001
4–7 cm T3a of FI	1071 (33.5)	226 (25.9)	845 (36.4)		5.87 [4.77–7.23]	<0.001	5.54 [4.48–6.85]^c^	<0.001
4–7 cm T3a of SI	997 (31.2)	98 (11.2)	899 (38.7)		14.4 [11.2–18.6]	<0.001	13.8 [10.5–17.9]^c^	<0.001
Sarcomatoid dedifferentiation, *N* (%)				0.003				
No	3053 (95.5)	849 (97.4)	2204 (94.8)		1 reference		1 reference	
Yes	143 (4.47)	23 (2.64)	120 (5.16)		2.01 [1.28–3.16]	<0.001	1.64 [1.00–2.70]	0.05
Fuhrman grade, *N* (%)				<0.001				
I/II	1631 (51.0)	514 (58.9)	1117 (48.1)		1 reference		1 reference	
III/IV	1565 (49.0)	358 (41.1)	1207 (51.9)		1.55 [1.33–1.82]	<0.001	1.17 [0.97–1.40]	0.094
Regional lymph nodes removed, *N* (%)				<0.001				
No	2841 (88.9)	836 (95.9)	2005 (86.3)					
Yes	355 (11.1)	36 (4.13)	319 (13.7)					

Abbreviations: CI, confidence interval; FI, perinephric‐fat invasion; IQR, interquartile range; OR, odds ratio; PR, partial nephrectomy; RCC, renal cell carcinoma; RN, radical nephrectomy; SI, sinus/perisinus invasion.

^a^ Adjust the covariables: sex, median household income, history of prior cancer, tumor size category, histology type, invasion type, sarcomatoid dedifferentiation, and Fuhrman grade.

^b^ Adjust the covariables: age, sex, median household income, history of prior cancer, histology type, invasion type, sarcomatoid dedifferentiation, and Fuhrman grade.

^c^ Adjust the covariables: age, sex, median household income, history of prior cancer, histology type, sarcomatoid dedifferentiation, and Fuhrman grade.

In our present study, a small proportion of patients with T3a RCCs (27.3%) underwent PN, and the proportion undergoing PN did not increase with a year of diagnosis (26.8% in 2010–2013, and 27.6% in 2014–2017; data not shown). A small proportion of patients with T3a RCC of sinus/perisinus extension underwent PN (10.4% for those with ≤4 cm T3a RCC of sinus/perisinus extension, and 11.2% for 4–7 cm T3a RCC of sinus/perisinus extension). Furthermore, the odds ratio (OR) values for performing RN versus PN were highest for patients with larger tumor diameter (4–7 cm) and T3a of sinus/perisinus extension pattern (OR = 4.50 [≤4 cm as reference], *p* < 0.001) and sinus/perisinus extension invasion pattern (OR = 3.45 [perinephric fat extension as reference], *p* < 0.001). In addition, age, year of diagnosis, sex, median household income status, history of other cancer, and sarcomatoid dedifferentiation were independent predictors for undergoing PN versus RN (Table 1).

Baseline comparison was conducted between the PN and RN cohorts stratified into four subgroups: 4–7 cm T3a RCC of perinephric fat extension, 4–7 cm T3a RCC of sinus/perisinus extension, ≤4 cm T3a RCC of perinephric fat extension, and ≤4 cm T3a RCC of sinus/perisinus extension. After IPTW adjustment, there was no significant difference between the PN and RN cohorts (detailed IPTW‐adjusted data analyses are shown in Tables S1 and S2).

### 3.2 Follow‐up and survival outcomes

**FIGURE 2:**
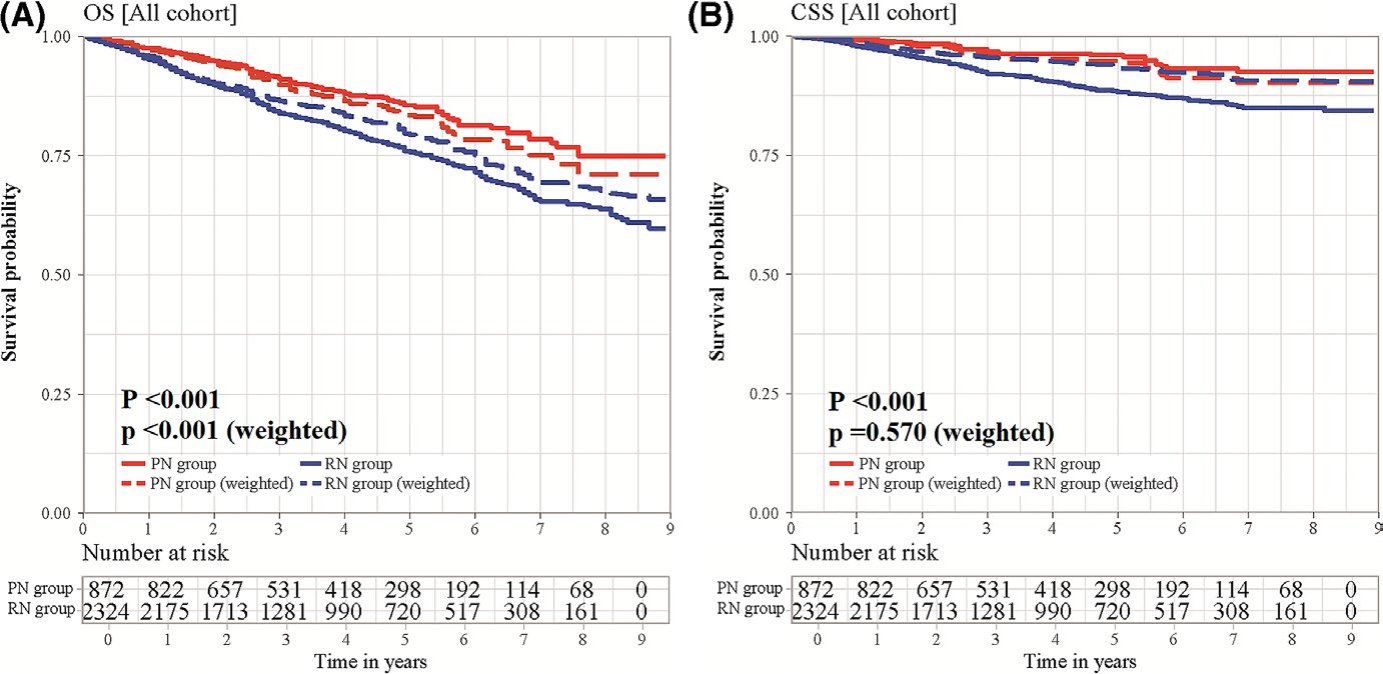
(A) Overall survival (OS) and (B) cancer‐specific survival (CSS) of patients with T3aN0/xM0 renal cell carcinoma (RCC) who received partial nephrectomy (PN) versus radical nephrectomy (RN), based on data with and without the inverse probability of treatment weighting (IPTW)

All patients had information of follow‐up data. In our present study, patients dead within 1 month or followed <1 month were excluded (Figure 1). Median follow‐up time was 4.25 years (range, 1 month to 8.9 years) for all patients, 4.33 years for the PN cohort, and 4.17 years for the RN cohort (log‐rank *p* = 0.18). In total, 34 patients (3.90%) in the PN group and 209 (8.99%) in the RN group died from RCC‐specific causes, while 78 (8.94%) and 292 (12.6%) patients died because of competing events in the PN and RN cohorts, respectively. Patients who obtained treatment of PN had a better OS than those receiving RN in all cohorts, regardless of IPTW adjustment (Figure 2). Furthermore, we analyzed data from 2010 to 2013, with a median 6.75‐year follow‐up, to compare OS and CSS between patients who underwent PN and RN, and our findings validated the above results; 5‐year OS rates were 83.6% and 74.2% for patients who received surgical treatment of PN and RN, respectively (Table 2). After IPTW adjustment, CSS was comparable in patients undergoing PN and RN in all cohorts and the four subgroups (Figure 2).

**TABLE 2:** Outcome analysis for PN versus RN in different subgroups

Subgroups	All causes mortality					Renal cell carcinoma special mortality^a^				
	5‐OS	HR (95% CI)	*p* value	Adjusted HR (95% CI)^b^	*p* value	5‐CSS	(S)HR (95% CI)	*p* value	Adjusted (S)HR (95% CI)^b^	*p* value
Complete data of 2010–2017										
All T3a RCC										
Partial nephrectomy	85.5%	1 reference		1 reference		96.0%	1 reference		1 reference	
Radical nephrectomy	75.8%	1.76 [1.43–2.16]	<0.001	1.56 [1.25–1.95]	<0.001	88.2%	2.35 [1.64–3.38]	<0.001	1.67 [1.12–2.50]	0.012
≤4 cm T3a of FI										
Partial nephrectomy	89.6%	1 reference		1 reference		99.0%	1 reference		1 reference	
Radical nephrectomy	77.2%	2.15 [1.48–3.13]	<0.001	1.98 [1.33–2.94]	0.001	91.6%	3.90 [1.72–8.85]	0.001	4.13 [1.66–10.28]	0.002
4–7 cm T3a of FI										
Partial nephrectomy	77.0%	1 reference		1 reference		91.0%	1 reference		1 reference	
Radical nephrectomy	73.3%	1.32 [0.95–1.84]	0.096	1.23 [0.87–1.74]	0.233	86.4%	1.51 [0.89–2.56]	0.12	1.11 [0.65–1.90]	0.700
≤4 cm T3a of SI										
Partial nephrectomy	87.3%	1 reference		1 reference		96.8%	1 reference		1 reference	
Radical nephrectomy	81.5%	1.72 [0.81–3.65]	0.157	1.62 [0.75–3.50]	0.224	93.1%	2.42 [0.56–10.5]	0.24	1.49 [0.32–7.01]	0.61
4–7 cm T3a of SI										
Partial nephrectomy	84.5%	1 reference		1 reference		92.7%	1 reference		1 reference	
Radical nephrectomy	76.0%	1.30 [0.77–2.21]	0.329	1.26 [0.73–2.18]	0.404	87.1%	1.13 [0.55–2.32]	0.73	0.98 [0.47–2.03]	0.95
Complete data of 2010–2013 (Sensitivity analyses)										
All T3a RCC										
Partial nephrectomy	83.6%	1 reference		1 reference		95.4%	1 reference		1 reference	
Radical nephrectomy	74.2%	1.62 [1.27–2.07]	<0.001	1.45 [1.11–1.89]	0.006	86.4%	2.32 [1.51–3.56]	<0.001	1.67 [1.02–2.75]	0.041
≤4 cm T3a of FI										
Partial nephrectomy	87.4%	1 reference		1 reference		98.6%	1 reference		1 reference	
Radical nephrectomy	78.1%	1.64 [1.06–2.53]	0.025	1.63 [1.04–2.56]	0.034	91.4%	2.86 [1.15–7.11]	0.023	2.94 [1.08–7.95]	0.034
4–7 cm T3a of FI										
Partial nephrectomy	73.3%	1 reference		1 reference		89.3%	1 reference		1 reference	
Radical nephrectomy	71.7%	1.22 [0.82–1.82]	0.325	1.19 [0.78–1.81]	0.416	84.7%	1.51 [0.78–2.91]	0.22	1.25 [0.62–2.51]	0.530
≤4 cm T3a of SI										
Partial nephrectomy	90.2%	1 reference		1 reference		96.7%	1 reference		1 reference	
Radical nephrectomy	76.9%	2.65 [0.94–7.45]	0.065	2.47 [0.87–6.98]	0.088	90.3%	3.50 [0.47–26.4]	0.22	3.43 [0.44–26.9]	0.240
4–7 cm T3a of SI										
Partial nephrectomy	81.1%	1 reference		1 reference		90.0%	1 reference		1 reference	
Radical nephrectomy	75.2%	0.99 [0.51–1.90]	0.973	1.07 [0.53–2.13]	0.859	85.0%	0.85 [0.38–1.94]	0.71	0.86 [0.37–1.99]	0.720
Inverse probability of treatment weighting PN vs. RN data										
All T3a RCC										
Partial nephrectomy	83.9%	1 reference		1 reference		94.8%	1 reference		1 reference	
Radical nephrectomy	78.0%	1.41 [1.12–1.77]	0.004	1.46 [1.16–1.84]	0.001	93.0%	1.00 [0.62–1.62]	0.990	1.03 [0.64–1.66]	0.890
≤4 cm T3a of FI										
Partial nephrectomy	88.0%	1 reference		1 reference		97.9%	1 reference		1 reference	
Radical nephrectomy	77.7%	1.62 [1.08–2.42]	0.02	1.74 [1.17–2.61]	0.007	96.2%	0.92 [0.34–2.48]	0.870	0.82 [0.32–2.09]	0.670
4–7 cm T3a of FI										
Partial nephrectomy	77.2%	1 reference		1 reference		91.0%	1 reference		1 reference	
Radical nephrectomy	76.4%	1.17 [0.82–1.67]	0.379	1.16 [0.81–1.66]	0.434	91.1%	1.22 [0.63–2.35]	0.55	1.31 [0.64–2.67]	0.46
≤4 cm T3a of SI										
Partial nephrectomy	87.3%	1 reference		1 reference		96.8%	1 reference		1 reference	
Radical nephrectomy	84.9%	1.36 [0.63–2.92]	0.431	1.27 [0.55–2.94]	0.579	98.3%	0.86 [0.14–5.43]	0.87	0.84 [0.09–7.73]	0.88
4–7 cm T3a of SI										
Partial nephrectomy	84.5%	1 reference		1 reference		92.7%	1 reference		1 reference	
adical nephrectomy	77.6%	1.22 [0.72–2.08]	0.462	1.21 [0.71–2.05]	0.488	90.8%	1.93 [0.78–4.81]	0.16	2.00 [0.76–5.26]	0.16
Propensity‐score‐matched data for PN cohort vs. RN cohort (Sensitivity analyses)										
All T3a RCC										
Partial nephrectomy	83.5%	1 reference		1 reference		94.8%	1 reference		1 reference	
Radical nephrectomy	78.3%	1.27 [0.97–1.66]	0.078	1.34 [1.03–1.76]	0.032	93.2%	1.09 [0.72–1.64]	0.695	1.16 [0.78–1.74]	0.464
≤4 cm T3a of FI										
Partial nephrectomy	87.2%	1 reference		1 reference		97.9%	1 reference		1 reference	
Radical nephrectomy	77.2%	1.69 [1.14–2.52]	0.009	1.78 [1.19–2.66]	0.005	96.1%	1.04 [0.44–2.46]	0.934	1.36 [0.55–3.39]	0.509
4–7 cm T3a of FI										
Partial nephrectomy	77.0%	1 reference		1 reference		91.1%	1 reference		1 reference	
Radical nephrectomy	76.2%	1.20 [0.80–1.82]	0.379	1.24 [0.81–1.89]	0.323	90.8%	1.02 [0.58–1.79]	0.941	1.08 [0.61–1.91]	0.797
≤4 cm T3a of SI										
Partial nephrectomy	87.3%	1 reference		1 reference		96.8%	1 reference		1 reference	
Radical nephrectomy	80.8%	1.71 [0.72–4.03]	0.224	1.50 [0.57–3.95]	0.414	96.8%	1.21 [0.28–5.27]	0.802	0.75 [0.12–4.66]	0.756
4–7 cm T3a of SI										
Partial nephrectomy	84.5%	1 reference		1 reference		92.7%	1 reference		1 reference	
Radical nephrectomy	71.4%	1.54 [0.78–3.06]	0.215	1.99 [0.94–4.18]	0.071	88.9%	0.98 [0.46–2.06]	0.951	0.77 [0.34–1.71]	0.518

Abbreviations: CI, confidence interval; CSS, cancer‐special survival; FI, perirenal fat invasion; OS, overall survival; PN, partial nephrectomy; RCC, renal cell carcinoma; RN, radical nephrectomy; (S)HR, (sub‐distribution) hazard ratio; SI, sinus/perisinus fat invasion.

^a^ Univariate and multivariate Fine and Gray proportional risk regression analysis to calculate the (S)HR (95% CI) was used for RCC‐special survival analysis in unweighted partial nephrectomy versus radical nephrectomy data and two sensitivity analyses; univariate and multivariate cause‐specific Cox regression analysis to calculate the HR (95% CI) was used for RCC‐special survival analysis in weighted partial nephrectomy versus radical nephrectomy data.

^b^ Adjust the covariables: age, sex, race, median household income, laterality, history of prior cancer, histology type, sarcomatoid dedifferentiation, and Fuhrman grade, regional lymph nodes removed.

### 3.3 Subgroup analysis

Since tumor diameter and extrarenal fat extension are two important risk factors influencing survival outcomes of patients with RCC,^15^, ^16^, ^21^ we further compared the prognosis of patients who received PN and RN in the four subgroups (Table 2). After adjustment for relevant covariables, we found that T3a RCC patients who underwent RN had a 1.56‐fold risk of all‐cause of death (adjusted HR: 1.56, 95% confidence interval [CI]: 1.25–1.95, *p* < 0.001), and 1.67‐fold risk of RCC‐specific mortality (adjusted HR: 1.67, 95% CI: 1.12–2.50, *p* = 0.012) relative to those who underwent PN. After IPTW adjustment, RN patients had a 1.46‐fold risk of all‐cause of death (adjusted HR: 1.46, 95% CI 1.16–1.84, *p* = 0.001) and a comparable risk of RCC‐specific mortality (adjusted HR: 1.03, 95% CI 0.64–1.66, *p* = 0.890) compared with PN, after adjusting for other covariables.

**FIGURE 3:**
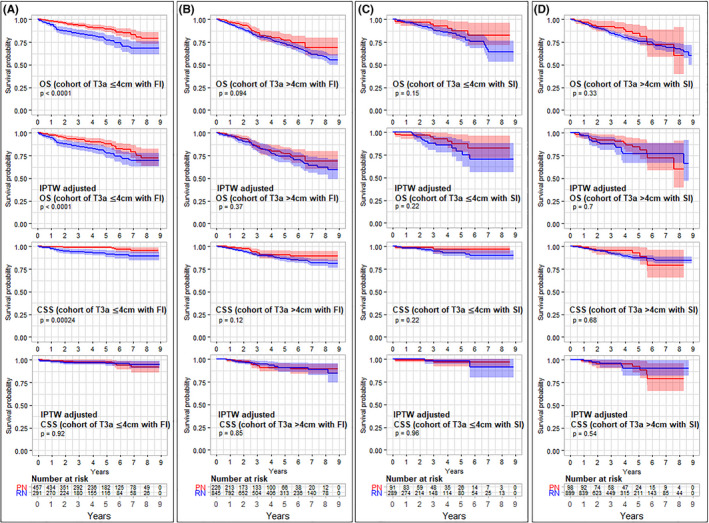
Overall survival (OS) and cancer‐specific survival (CSS) of patients with T3aN0/xM0 renal cell carcinoma (RCC) who underwent partial nephrectomy (PN) versus radical nephrectomy (RN) based on data with and without the inverse probability of treatment weighting (IPTW). Results from patients with (A) 4–7 cm T3a RCC of perirenal fat invasion (FI); (B) ≤4 cm T3a RCC of FI; (C) ≤4 cm T3a RCC of sinus/perisinus fat invasion (SI); and (D) 4–7 cm T3a RCC of SI

Inverse probability of treatment weighting data analysis indicated a comparable CSS between the PN and RN cohorts for all four subgroups (T3a ≤4 cm with perinephric fat extension, T3a 4–7 cm with perinephric fat extension, T3a ≤4 cm with Sinus/perisinus extension, and T3a 4–7 cm with Sinus/perisinus extension). In addition, PN did not result in significantly better OS relative to RN in the T3a 4–7 cm with perinephric fat extension, T3a ≤4 cm with sinus/perisinus extension, and T3a 4–7 cm with sinus/perisinus extension subgroups (Figure 3, Table 2); however, PN was associated with significantly improved OS in the T3a ≤4 cm with perinephric fat extension subgroup (5‐year OS: 88.0% vs. 77.7%; adjusted HR: 1.74, 95% CI: 1.17–2.61, *p* = 0.007; Table 3). Sensitivity analyses generated similar results, validating these findings (Table 2). In addition, similar results were observed after propensity‐score matching analysis which did not include the covariables of postoperative (data not shown).

**TABLE 3:** Overview of the current literature regarding partial nephrectomy and radical nephrectomy in patients with pathological T3a renal cell carcinoma

Study	Cohort (PN/RN)	Tumor size, cm (PN/RN)	Follow‐up, months	Results
Ramaswamy et al.^4^	44/22	3.8^c^, ^a^	50^c^	1. Larger tumors, clear cell histology, and positive surgical margins were at the greatest risk of upstaging 2. No patients had a recurrence or RCC‐specific mortality in PN and RN cohorts
Oh et al.^10^ ^,^ ^b^	45/298	3.50 (1.55)/7.99 (3.68)^a^	43^a^	For pT3a RCC, 2.2%, 0.0%, and 4.4% vs. 18.5%, 12.8%, and 31.5% of patients with all‐ and RCC‐caused mortality, and recurrence at a median follow‐up of 44 and 43 months for PN and RN, respectively
Shvero et al.^11^ ^,^ ^c^	48/86	4.0 (2.8–5.2)/7.0 (5–9.5)	55.2 for PN vs. 48.8 for RN	1. The surgery type was not associated with local recurrence (*p* = 0.978), metastatic progression (*p* = 0.972), disease‐specific survival (*p* = 0.626), or overall survival (*p* = 0.974)
Lee et al.^12^	57/158	5.0 (3.7–6.2)^c^	–	1. Patients upstaged from clinical stage T1 to pathologic stage T3a RCC showed shorter survival outcomes than those without upstaging 2. No significant differences in recurrence‐specific survival, overall survival, and disease‐specific survival between PN and RN
Shah et al.^14^	49/91	‐	37^c^	1. Larger tumor size was associated with a higher risk of pT3a upstaging (4.4% vs. 24.4% upstaged from clinical stage T1a vs. clinical stage T1b) 2. Positive surgical margin increased the risk of recurrence, 31% of pT3a patients experience recurrence during a median follow‐up of 38 months; median time to recurrence was 18 months 3. Shorter recurrence‐specific survival was observed after PN compared with RN because positive surgical margin was observed only for PN
Weight et al.^24^	66/80	‐	53 (3–72)^c^	PN exhibited a better disease‐specific survival than RN in Kaplan–Meier analyses.
Andrade et al.^25^	70/70	‐	‐	1. 2.9% and 1.4% of patients had local recurrence after robotic‐assisted PN and RN 2. 8.6% and 5.7% of patients had distant metastasis after robotic‐assisted PN and RN 3. Robotic‐assisted PN vs. RN: 3‐year overall survival, disease‐specific survival, and recurrence‐specific survival was 9% vs. 84%, 94% vs. 95%, and 95% vs. 10%, respectively (all p values were non‐significant)
Nayak et al.^27^	66/68	‐	23^c^	The 3‐year recurrence‐specific survival was 73% for PN vs. 77% for RN
Capitanio et al.^28^ ^,^ ^b^	71/238	3.0 (2.2–4.4)/5.5 (4.2–6.5)	55 for RN vs. 43 for PN^a^	1. 2.9% vs. 2.8% of cases experienced local recurrence in PN vs. RN 2. PN cohort: 1‐, 2‐, and 5‐year metastatic progression was 9.1%, 13.3%, and 24.1%, respectively; PN cohort: 1‐, 2‐, and 5‐year cancer‐specific mortality was 3.5%, 10.7%, and 18.4%, respectively 3. There were no differences in metastatic progression and cancer‐specific mortality between PN and RN after propensity‐score matching
Peng et al.^29^	18/18	5.27 (1.5)/5.03 (1.42)^a^	35.5 (10–86)^c^	1. The 5‐year disease‐specific survival and recurrence‐specific survival for PN and RN patients was 80.5% vs. 85.9%, respectively, (*p* = 0.305) and 76% vs. 80.8%, respectively, (*p* = 0.524) 2. Cox multivariate regression analysis showed that the surgery type (RN vs. PN) was not associated significantly with disease‐specific survival or recurrence‐specific survival
Jeong et al.^30^	37/54	‐	48.5 (27.8)^c^, ^a^	PN and RN showed no significant difference in 2‐year recurrence‐specific survival (91.9% vs. 83.7%, respectively, *p* = 0.251)

Note

Tumor size and follow‐up time are presented as the median (IQR).

Abbreviations: IQR, interquartile range; PN, partial nephrectomy; RCC, renal cell carcinoma; RN, radical nephrectomy.

^a^ Data from two institutions.

^b^ Data are the mean or mean (standard deviation).

^c^ Data from multiple institutions.

## 4 DISCUSSION

Early detection of small RCC (particularly T1a) tumors favors the adoption of treatment by PN.^1^ Clinically, T1 RCC tumors can be identified as more aggressive T3a masses after final pathology analysis post‐PN, with prior publications reporting a 1.9%–14% incidence rate.^7^, ^8^, ^9^ In large RCC tumors, extrarenal fat extension is usually grossly visible on computed tomography or magnetic resonance imaging, whereas microscopic examination is generally required for small tumors.^23^ Thus, the vast majority of pathological T3a RCC tumors, upstaged from the clinical T1 stage, need a pathological diagnosis and cannot be detected by preoperative imaging.

Beksac et al.^9^ observed that larger tumor diameter and older age were two significant risk factors associated with clinical T1 RCC pathological upstaging to T3a RCC post‐PN based on a high‐volume multicenter cohort and national registry data. A recent meta‐analysis by Veccia et al.^5^ summarized the predictors of risk for pathological T3a upstaging from clinical T1 RCC and found that the characteristics, older age, larger tumor diameter, and higher RCC complexity, increased the risk of postoperative upstaging. Further, clear cell histology is also associated with a higher risk of histology upstage.^4^ Another study indicated that RCC diameter, R.E.N.A.L. score, and systemic inflammatory response markers (such as lymphocyte to monocyte ratio) can be used to predict postoperative T3a upstaging^7^; however, more accurate and better characterization of RCC is needed to predict preoperative T3a RCC upstaging.

Indeed, the dilemma generated by upstaging to a more aggressive pathological T3a tumor postoperatively could jeopardize patient survival and lead to challenges for treatment decisions after primary PN.^5^ In our present study, compared to RN, PN can result in better, or at least a comparable, OS and CSS for T3a RCC. These results are consistent with those of a prior meta‐analysis.^8^ Besides, the majority of studies show that PN of small T3aN0M0 masses does not yield an inferior prognosis^4^, ^10^, ^11^, ^12^, ^14^, ^24^, ^25^, ^26^, ^27^, ^28^, ^29^, ^30^, ^31^, ^32^ (partial results presented in Table 3). Although the findings of our present study may not be useful for preoperative “surgical planning”, our data support that it is oncologically safe to perform PN for small renal masses, even if the tumor is subsequently pathologically determined to be T3a, rather than T1.

Prior results from the SEER database showed that cardiovascular diseases were the main cause of non‐RCC special mortality, especially for patients with localized RCCs after 5 years of treatment.^33^ PN contributed to the prevention of the risk of cardiovascular diseases, which is associated with improved OS,^34^ preservation of glomerular filtration rate related to PN might be considered as a potential explanation for the observation.^35^, ^36^ However, such effects might be based on the tumor size of RCC, the mortality by cardiovascular after RN was increased for RCC <2 cm, besides,^37^ for small T1N0M0 RCC more than 4 cm, there was no significant difference in cardiovascular diseases incidence for RN versus PN.^33^ Compared with large‐diameter T3a RCC after PN, the small diameter T3a RCC obtains more normal renal parenchymal retention and has a more prominent role in the protection of renal function, and these patients benefit more. For complex small T3a RCCs, for example, T3 endophytic might require a longer operative time and deep suture, which might impact kidney function. But because of the application of robot‐assisted PN, the preoperative evaluation of imaging three‐dimensional imaging technology, and the increased experience of the surgeon, the warm ischemia time is shortened compared with the past, which may not affect the postoperative renal function.^3^, ^31^, ^38^, ^39^

In complex RCC, to achieve trifecta after PN, surgical technique plays an important role.^40^ Previous studies have reported that surgical technique is an independent predictor of trifecta outcome. The compliance rate of Trifecta outcome in open PN, laparoscopic‐assisted PN, and robot‐assisted PN was 49%, 50.6%, and 69.9% (*p* = 0.003). A recent meta‐analysis concluded that for complex RCC, compared to laparoscopic PN, robot‐assisted PN can achieve a lower conversion rate of open surgery, shorter warm ischemia time, and better renal function protection than laparoscopic PN.^39^ It is also necessary to pay attention to the influence of the methodological limitations of observational research on the results. For T3a RCC, it was previously reported that robot‐assisted PN technology can achieve negative margins in T3a RCC patients with sinus fat extension, and achieve good tumor control in a short follow‐up time.^31^ However, a longer follow‐up is needed, so as to better evaluate the effect of robot‐assisted PN on the long‐term prognosis of pT3a tumors. In addition, the study did not compare with other surgical methods, so it is difficult to describe the superiority of this technique. In addition, this study also emphasizes that the surgeon's experience and the complexity of RCC play an important role in predicting the outcome of surgery.

Prior studies indicate that patients with RCC tumors with a larger diameter, or classified as T3a with sinus/perisinus extension, experience worse prognosis than those with small or T3a with perinephric fat extension tumors^15^, ^16^, ^21^, ^41^; therefore, analyses of survival outcomes should consider tumor diameter and extension pattern. Here we report an important finding, that PN improved OS relative to RN for RCC patients with tumors ≤4 cm classified as T3aN0/xM0 with perinephric fat extension, and is associated with comparable OS for those with T3aN0/xM0 RCC of 4–7 cm with perinephric fat extension or sinus/perisinus extension across all tumor diameters, while PN led to comparable CSS to RN in all four subgroups.

Local recurrence is a relevant issue for patients with T3a RCC; however, it remains controversial. In a retrospective study, Lee et al.^12^ observed that approximately 6.3% (215/3431) of patients with T1 RCC underwent pathological upstaging to T3a stage and that there were no significant differences in recurrence‐free survival or OS between the PN and RN cohorts, and Oh et al. observed similar results.^10^ Nevertheless, Chevinsky et al.^13^ reported that pathological stage T3a significantly shortened recurrence‐free survival; however, they included T3a pathologically upstaged from larger size T2 and T3a RCC with renal vein extension in their study. Notably, a recent meta‐analysis proved that PN and RN are associated with similar recurrence‐free survival.^8^ The survival outcomes of patients with T3aN0/xM0 RCC may be determined mostly by inherent cancer biological features, rather than whether surgical PN or RN is applied. Hence, upstaging to T3aN0M0 RCC could not prevent surgeons from conducting PN.

In addition, it should be noted that positive surgical margins can increase the risk of local recurrence.^42^ For T3a RCC, the incidence of positive surgical margins for PN is higher, with an average of about 18%.^31^ Although the impact of positive surgical margins after PN on the prognosis is a matter of debate, some prior studies observed positive surgical margins were significantly associated with aggressive disease characteristics and low surgeon experience,^38^ but not significantly impact the short‐term survival functions.^38^, ^43^

Our study had some unavoidable limitations. Although the SEER database has the advantages of its large sample size, it also has some drawbacks, including the inherent limitation of its retrospective nature. The inability to adequately control the selection bias between the PN and RN cohorts is the main limitation of the study. Although the method of IPTW and propensity score matching was used in the current study, the SEER analysis does lack sufficient confounding information (such as patient performance status, body mass index, comorbidities, renal function, renal vein invasion, time from diagnosis to surgery, surgeon skill, and institutional case volume) to adequately control the selection factors that affect survival outcomes. Our study identified patients with pathologically confirmed ≤7 cm T3aN0/xM0 RCC (assumed that those patients who received PN were clinical T1 but upgraded to T3aN0/xM0 RCC after pathological examination). However, we found that because PN is the preferred treatment for T1 RCC, those who receive RN may have a higher stage than T1. Therefore, those who receive RN are more likely to be at an advanced stage than those who receive PN. This imbalance between the two stages cannot be solved by statistical methods such as IPTW and propensity score matching. Because there is no higher level of evidence, a more rigorously designed randomized controlled study to prove the prognostic difference between the two surgical methods PN and RN in T3a RCC, our study is an addition to the previous literature, even though our study has limitations.

## 5 CONCLUSIONS

Our present study shows that, relative to RN, PN provided comparable CSS, and even improved OS in patients with ≤7 cm T3aN0/xM0 RCC of only extrarenal fat extension. Our study indicated that the treatment of PN is a preferred treatment for clinical T1 renal mass, even where there is pathological upstaging to T3a RCC. The pathological characteristic of extrarenal fat extension pattern and tumors size does not shorten the survival benefit of PN. Nevertheless, there is no high‐level evidence to validate our findings or prior similar studies; therefore, these results should be interpreted with caution, and further validation in multicenter randomized‐design studies with large sample sizes and longer follow‐up is warranted.

## CONFLICT OF INTEREST

There is no conflict interest.

## Supporting information

TableS1‐S2

## Data Availability

All detailed data can be downloaded using SEER *Stat software after registration for access to the SEER database.
