# Supplementary material for: Comparison of prognosis between patients undergoing radical nephrectomy versus partial nephrectomy for renal cell carcinoma ≤7 cm T3aN0/xM0: Survival benefit is biased toward partial nephrectomy
Source: Cancer Med. 2021 Nov 14;10(24):8909–23. doi: 10.1002/cam4.4412 (PMC8683550; doi:10.1002/cam4.4412)
Supplement: Supplementary file 1 — TableS1‐S2 [file CAM4-10-8909-s001.docx]

| Supplementary Table 1: Comparison between PN and RN patients after inverse probability of treatment weighting. | | | | | | | | | | | | | | | | | | | | | | | | | | | | | | | | | | | | | | | | |  |
| --- | --- | --- | --- | --- | --- | --- | --- | --- | --- | --- | --- | --- | --- | --- | --- | --- | --- | --- | --- | --- | --- | --- | --- | --- | --- | --- | --- | --- | --- | --- | --- | --- | --- | --- | --- | --- | --- | --- | --- | --- | --- |
|  | **All cohort** | | | |  | | **T3a ≤4cm with FI** | | | | | | |  | | **T3a 4-7cm with FI** | | | | | | |  | | **T3a ≤cm with SI** | | | | | | |  | | **T3a 4-7cm with SI** | | | | | | | |
|  | **Overall** | **PN** | **RN** | **p value** | | **Overall** | | **PN** | | **RN** | | **p value** | | | **Overall** | | **PN** | | **RN** | | **p value** | | | **Overall** | | **PN** | | **RN** | | **p value** | | | **Overall** | | **PN** | | **RN** | | **p value** | |  |
| **Year at diagnosis, %** |  |  |  |  |  | |  | |  | |  | |  |  | |  | |  | |  | |  |  | |  | |  | |  | |  |  | |  | |  | |  | |  | |
| 2010-2013 | 43.5 | 43.6 | 43.5 | 0.952 |  | | 52.6 | | 52.9 | | 52.3 | | 0.891 |  | | 40.6 | | 40.6 | | 40.6 | | 0.987 |  | | 34.5 | | 34.1 | | 35 | | 0.872 |  | | 32.5 | | 32.7 | | 32.4 | | 0.962 | |
| 2014-2017 | 56.5 | 56.4 | 56.5 |  |  | | 47.4 | | 47.1 | | 47.7 | |  |  | | 59.4 | | 59.4 | | 59.4 | |  |  | | 65.5 | | 65.9 | | 65 | |  |  | | 67.5 | | 67.3 | | 67.6 | |  | |
| **Age at diagnosis,median [IQR]** | 65.00 [56.00, 72.00] | 65.00 [57.00, 72.00] | 65.00 [56.00, 73.00] | 0.391 |  | | 66.00 [57.64, 73.00] | | 66.00 [58.00, 73.00] | | 67.00 [57.00, 74.00] | | 0.384 |  | | 65.00 [58.00, 72.00] | | 64.00 [58.00, 72.00] | | 66.00 [57.00, 73.00] | | 0.549 |  | | 64.00 [54.67, 71.00] | | 64.00 [54.00, 70.00] | | 64.00 [55.00, 71.00] | | 0.906 |  | | 64.00 [56.00, 72.00] | | 64.00 [56.50, 72.00] | | 63.00 [56.00, 72.00] | | 0.995 | |
| **Age at dignosis (category), %** |  |  |  |  |  | |  | |  | |  | |  |  | |  | |  | |  | |  |  | |  | |  | |  | |  |  | |  | |  | |  | |  | |
| 18-60 year | 35.6 | 35.7 | 35.4 | 0.913 |  | | 31.1 | | 31.6 | | 30.6 | | 0.796 |  | | 33 | | 33.3 | | 32.8 | | 0.894 |  | | 37.4 | | 37.4 | | 37.5 | | 0.986 |  | | 40.8 | | 40.8 | | 40.8 | | 1 | |
| 60-85year | 64.4 | 64.3 | 64.6 |  |  | | 68.9 | | 68.4 | | 69.4 | |  |  | | 67 | | 66.7 | | 67.2 | |  |  | | 62.6 | | 62.6 | | 62.5 | |  |  | | 59.2 | | 59.2 | | 59.2 | |  | |
| **Race, N (%)** |  |  |  |  |  | |  | |  | |  | |  |  | |  | |  | |  | |  |  | |  | |  | |  | |  |  | |  | |  | |  | |  | |
| White | 84.4 | 84.6 | 84.3 | 0.971 |  | | 83.8 | | 83.9 | | 83.8 | | 0.999 |  | | 83.6 | | 84.2 | | 83.1 | | 0.942 |  | | 85.5 | | 85.7 | | 85.3 | | 0.993 |  | | 90.8 | | 90.8 | | 90.7 | | 1 | |
| Black | 8.6 | 8.4 | 8.7 |  |  | | 10.8 | | 10.7 | | 10.8 | |  |  | | 8.9 | | 8.6 | | 9.3 | |  |  | | 7.9 | | 7.7 | | 8.1 | |  |  | | 4.1 | | 4.1 | | 4.1 | |  | |
| Other | 7 | 7 | 7 |  |  | | 5.4 | | 5.4 | | 5.4 | |  |  | | 7.4 | | 7.2 | | 7.6 | |  |  | | 6.6 | | 6.6 | | 6.6 | |  |  | | 5.1 | | 5.1 | | 5.2 | |  | |
| **Sex, N (%)** |  |  |  |  |  | |  | |  | |  | |  |  | |  | |  | |  | |  |  | |  | |  | |  | |  |  | |  | |  | |  | |  | |
| Female | 29.8 | 29.5 | 30.2 | 0.76 |  | | 31.2 | | 31 | | 31.4 | | 0.923 |  | | 22.1 | | 21.7 | | 22.4 | | 0.836 |  | | 39.1 | | 39.6 | | 38.6 | | 0.87 |  | | 43.9 | | 43.9 | | 44 | | 0.989 | |
| Male | 70.2 | 70.5 | 69.8 |  |  | | 68.8 | | 69 | | 68.6 | |  |  | | 77.9 | | 78.3 | | 77.6 | |  |  | | 60.9 | | 60.4 | | 61.4 | |  |  | | 56.1 | | 56.1 | | 56 | |  | |
| **Median household income, %** |  |  |  |  |  | |  | |  | |  | |  |  | |  | |  | |  | |  |  | |  | |  | |  | |  |  | |  | |  | |  | |  | |
| Less than $59999 | 34.4 | 34.5 | 34.4 | 0.969 |  | | 39.5 | | 40 | | 39.1 | | 0.812 |  | | 34.6 | | 34.6 | | 34.6 | | 0.99 |  | | 36.5 | | 36.3 | | 36.6 | | 0.949 |  | | 27.4 | | 27.6 | | 27.3 | | 0.962 | |
| More than $60,000 | 65.6 | 65.5 | 65.6 |  |  | | 60.5 | | 60 | | 60.9 | |  |  | | 65.4 | | 65.4 | | 65.4 | |  |  | | 63.5 | | 63.7 | | 63.4 | |  |  | | 72.6 | | 72.4 | | 72.7 | |  | |
| **With prior other cancer, %** |  |  |  |  |  | |  | |  | |  | |  |  | |  | |  | |  | |  |  | |  | |  | |  | |  |  | |  | |  | |  | |  | |
| No | 77.7 | 78 | 77.4 | 0.742 |  | | 79.5 | | 80.1 | | 78.9 | | 0.711 |  | | 72.3 | | 73.3 | | 71.3 | | 0.584 |  | | 80.6 | | 80.2 | | 80.9 | | 0.886 |  | | 78.6 | | 78.6 | | 78.7 | | 0.985 | |
| Yes | 22.3 | 22 | 22.6 |  |  | | 20.5 | | 19.9 | | 21.1 | |  |  | | 27.7 | | 26.7 | | 28.7 | |  |  | | 19.4 | | 19.8 | | 19.1 | |  |  | | 21.4 | | 21.4 | | 21.3 | |  | |
| **Laterality, N (%)** |  |  |  |  |  | |  | |  | |  | |  |  | |  | |  | |  | |  |  | |  | |  | |  | |  |  | |  | |  | |  | |  | |
| Left | 49.5 | 49.4 | 49.6 | 0.917 |  | | 48.9 | | 48.6 | | 49.2 | | 0.876 |  | | 52.3 | | 52.7 | | 51.8 | | 0.816 |  | | 45.6 | | 45.1 | | 46.1 | | 0.873 |  | | 48.2 | | 48 | | 48.5 | | 0.92 | |
| Right | 50.5 | 50.6 | 50.4 |  |  | | 51.1 | | 51.4 | | 50.8 | |  |  | | 47.7 | | 47.3 | | 48.2 | |  |  | | 54.4 | | 54.9 | | 53.9 | |  |  | | 51.8 | | 52 | | 51.5 | |  | |
| **Size, median [IQR]** | 4.00 [3.10, 5.00] | 4.00 [3.10, 5.00] | 4.00 [3.00, 5.00] | 0.933 |  | | 3.10 [2.50, 3.70] | | 3.10 [2.50, 3.70] | | 3.20 [2.50, 3.60] | | 0.639 |  | | 5.00 [4.50, 5.70] | | 5.00 [4.50, 5.80] | | 5.00 [4.50, 5.70] | | 0.506 |  | | 3.10 [2.50, 3.50] | | 3.10 [2.50, 3.50] | | 3.20 [2.50, 3.60] | | 0.684 |  | | 5.11 [4.50, 5.90] | | 5.10 [4.55, 5.80] | | 5.11 [4.50, 6.00] | | 0.993 | |
| **Size (category), %** |  |  |  |  |  | |  | |  | |  | |  |  | |  | |  | |  | |  |  | |  | |  | |  | |  |  | |  | |  | |  | |  | |
| ≦4cm | 51.9 | 51.4 | 52.3 | 0.706 |  | |  | |  | |  | |  |  | |  | |  | |  | |  |  | |  | |  | |  | |  |  | |  | |  | |  | |  | |
| 4-7cm | 48.1 | 48.6 | 47.7 |  |  | |  | |  | |  | |  |  | |  | |  | |  | |  |  | |  | |  | |  | |  |  | |  | |  | |  | |  | |
| **Histological type, %** |  |  |  |  |  | |  | |  | |  | |  |  | |  | |  | |  | |  |  | |  | |  | |  | |  |  | |  | |  | |  | |  | |
| Clear cell RCC | 63.9 | 63.9 | 63.9 | 0.985 |  | | 60.5 | | 60.7 | | 60.3 | | 0.925 |  | | 57.3 | | 57.9 | | 56.6 | | 0.74 |  | | 73.7 | | 73.6 | | 73.7 | | 0.983 |  | | 81.4 | | 81.6 | | 81.1 | | 0.895 | |
| Other/undefined | 36.1 | 36.1 | 36.1 |  |  | | 39.5 | | 39.3 | | 39.7 | |  |  | | 42.7 | | 42.1 | | 43.4 | |  |  | | 26.3 | | 26.4 | | 26.3 | |  |  | | 18.6 | | 18.4 | | 18.9 | |  | |
| **Pathological T3a invasion type, %** |  |  |  |  |  | |  | |  | |  | |  |  | |  | |  | |  | |  |  | |  | |  | |  | |  |  | |  | |  | |  | |  | |
| Perinephric fat invasion | 71.5 | 71.7 | 71.3 | 0.827 |  | |  | |  | |  | |  |  | |  | |  | |  | |  |  | |  | |  | |  | |  |  | |  | |  | |  | |  | |
| Sinus/perisinus fat invasion | 28.5 | 28.3 | 28.7 |  |  | |  | |  | |  | |  |  | |  | |  | |  | |  |  | |  | |  | |  | |  |  | |  | |  | |  | |  | |
| **Group, %** |  |  |  |  |  | |  | |  | |  | |  |  | |  | |  | |  | |  |  | |  | |  | |  | |  |  | |  | |  | |  | |  | |
| T3a≦4cm and FI | 38.2 | 37.9 | 38.4 | 0.974 |  | |  | |  | |  | |  |  | |  | |  | |  | |  |  | |  | |  | |  | |  |  | |  | |  | |  | |  | |
| T3a≦4cm and SI | 13.7 | 13.5 | 13.9 |  |  | |  | |  | |  | |  |  | |  | |  | |  | |  |  | |  | |  | |  | |  |  | |  | |  | |  | |  | |
| T3a 4-7cm and FI | 33.4 | 33.8 | 32.9 |  |  | |  | |  | |  | |  |  | |  | |  | |  | |  |  | |  | |  | |  | |  |  | |  | |  | |  | |  | |
| T3a 4-7cm and SI | 14.8 | 14.7 | 14.8 |  |  | |  | |  | |  | |  |  | |  | |  | |  | |  |  | |  | |  | |  | |  |  | |  | |  | |  | |  | |
| **Sarcomatoid dedifferentiation, %** |  |  |  |  |  | |  | |  | |  | |  |  | |  | |  | |  | |  |  | |  | |  | |  | |  |  | |  | |  | |  | |  | |
| No | 96.9 | 96.9 | 96.9 | 0.941 |  | | 98 | | 97.9 | | 98.1 | | 0.915 |  | | 96.3 | | 96.4 | | 96.2 | | 0.881 |  | | 98.9 | | 98.9 | | 98.9 | | 0.973 |  | | 94.7 | | 94.9 | | 94.6 | | 0.904 | |
| Yes | 3.1 | 3.1 | 3.1 |  |  | | 2 | | 2.1 | | 1.9 | |  |  | | 3.7 | | 3.6 | | 3.8 | |  |  | | 1.1 | | 1.1 | | 1.1 | |  |  | | 5.3 | | 5.1 | | 5.4 | |  | |
| **Fuhrman grade, %** |  |  |  |  |  | |  | |  | |  | |  |  | |  | |  | |  | |  |  | |  | |  | |  | |  |  | |  | |  | |  | |  | |
| I/II | 55.4 | 55.8 | 55 | 0.738 |  | | 62.6 | | 62.7 | | 62.5 | | 0.969 |  | | 54.4 | | 54.8 | | 53.9 | | 0.83 |  | | 55.2 | | 56 | | 54.4 | | 0.791 |  | | 37 | | 36.7 | | 37.4 | | 0.907 | |
| III/IV | 44.6 | 44.2 | 45 |  |  | | 37.4 | | 37.3 | | 37.5 | |  |  | | 45.6 | | 45.2 | | 46.1 | |  |  | | 44.8 | | 44 | | 45.6 | |  |  | | 63 | | 63.3 | | 62.6 | |  | |
| **Regional lymph nodes removed, %** |  |  |  |  |  | |  | |  | |  | |  |  | |  | |  | |  | |  |  | |  | |  | |  | |  |  | |  | |  | |  | |  | |
| No | 94.5 | 94.6 | 94.3 | 0.766 |  | | 95.2 | | 95.4 | | 95.1 | | 0.896 |  | | 93.2 | | 93.2 | | 93.2 | | 0.999 |  | | 96.7 | | 96.7 | | 96.7 | | 0.984 |  | | 92.8 | | 92.9 | | 92.8 | | 0.97 | |
| Yes | 5.5 | 5.4 | 5.7 |  |  | | 4.8 | | 4.6 | | 4.9 | |  |  | | 6.8 | | 6.8 | | 6.8 | |  |  | | 3.3 | | 3.3 | | 3.3 | |  |  | | 7.2 | | 7.1 | | 7.2 | |  | |
| **Median time to event/censor, median [IQR]. years** | 3.67 [1.92, 5.69] | 3.83 [2.00, 5.58] | 3.50 [1.92, 5.83] | 0.229 |  | | 4.25 [2.17, 6.25] | | 4.42 [2.25, 6.33] | | 4.17 [2.09, 6.20] | | 0.575 |  | | 3.33 [2.08, 5.50] | | 3.50 [2.25, 5.42] | | 3.25 [2.00, 5.74] | | 0.646 |  | | 3.08 [1.83, 5.08] | | 3.17 [1.58, 5.13] | | 3.08 [1.92, 5.08] | | 0.454 |  | | 3.25 [1.75, 4.92] | | 3.92 [1.96, 4.92] | | 2.83 [1.58, 4.75] | | 0.026 | |
| **Estimated median follow-up time, years** | 4.33 | 4.33 | 4.25 | 0.18 |  | | 4.92 | | 4.92 | | 5.17 | | 0.052 |  | | 4.25 | | 4.33 | | 4.17 | | <0.001 |  | | 3.50 | | 3.42 | | 3.58 | | 0.11 |  | | 3.83 | | 4.17 | | 3.33 | | 0.74 | |
| **Survival outcomes, %** |  |  |  |  |  | |  | |  | |  | |  |  | |  | |  | |  | |  |  | |  | |  | |  | |  |  | |  | |  | |  | |  | |
| Alive | 82.7 | 85.5 | 79.9 | 0.009 |  | | 82.6 | | 86.6 | | 78.7 | | 0.011 |  | | 79.8 | | 81.4 | | 78.2 | | 0.475 |  | | 89.4 | | 91.2 | | 87.6 | | 0.623 |  | | 84 | | 84.7 | | 83.2 | | 0.675 | |
| Dead from RCC | 5.9 | 4.8 | 7.1 |  |  | | 4.9 | | 2.4 | | 7.3 | |  |  | | 7 | | 7 | | 7 | |  |  | | 2.5 | | 2.2 | | 2.7 | |  |  | | 7.6 | | 8.2 | | 7.1 | |  | |
| Dead from other events | 11.4 | 9.7 | 13 |  |  | | 12.5 | | 11 | | 14 | |  |  | | 13.2 | | 11.6 | | 14.9 | |  |  | | 8.1 | | 6.6 | | 9.7 | |  |  | | 8.4 | | 7.1 | | 9.7 | |  | |
| RCC: renal cell carcinoma; PN: Partial nephrectomy; RN: Radical nephrectomy; OR: odds ratio; IQR: interquartile range; CI: confidence interval; FI: perirenal fat invasion; SI: sinus/perisinus fat invasion; | | | | | | | | | | | | | | | | | | | | | | | | | | | | | | | | | | | | | | | | |  |

| Supplementary Table 2: Comparison between PN and RN patients before and after the inverse probability of treatment weighting. | | | | | | | | | | | | | | | | | | | | | | | |
| --- | --- | --- | --- | --- | --- | --- | --- | --- | --- | --- | --- | --- | --- | --- | --- | --- | --- | --- | --- | --- | --- | --- | --- |
|  | **T3a ≤4cm with FI** | | | |  | **T3a 4-7cm with FI** | | | |  | **T3a ≤4cm with SI** | | | | |  | | **T3a 4-7cm with SI** | | | | |  |
|  | **PN group** | **RN group** | **P** | **P weighted** |  | **PN group** | **RN group** | **P** | **P weighted** |  | **PN group** | **RN group** | **P** | **P weighted** |  | | **PN group** | | **RN group** | **P** | **P weighted** |  |  |
| **N** | 457 | 291 |  |  |  | 226 | 845 |  |  |  | 91 | 289 |  |  |  | | 98 | | 899 |  |  |  |  |
| **Year at diagnosis, N (%)** |  |  | 0.221 | 0.891 |  |  |  | 0.001 | 0.987 |  |  |  | 0.534 | 0.872 |  | |  | |  | 1.000 | 0.962 |  |  |
| 2010-2013 | 218 (47.7) | 153 (52.6) |  |  |  | 90 (39.8) | 449 (53.1) |  |  |  | 31 (34.1) | 111 (38.4) |  |  |  | | 32 (32.7) | | 298 (33.1) |  |  |  |  |
| 2014-2017 | 239 (52.3) | 138 (47.4) |  |  |  | 136 (60.2) | 396 (46.9) |  |  |  | 60(65.9) | 178 (61.6) |  |  |  | | 66 (67.3) | | 601 (66.9) |  |  |  |  |
| **Age at diagnosis, median [IQR]** | 63.0 [54.0;70.0] | 68.0 [59.0;74.5] | <0.001 | 0.384 |  | 64.0 [58.0;72.0] | 66.0 [58.0;73.0] | 0.089 | 0.549 |  | 64.0 [54.5;70.0] | 64.0 [56.0;73.0] | 0.425 | 0.906 |  | | 64.0 [57.0;72.0] | | 65.0 [57.0;72.0] | 0.598 | 0.995 |  |  |
| **Age at diagnosis (category), N (%)** |  |  | <0.001 | 0.796 |  |  |  | 0.437 | 0.894 |  |  |  | 0.956 | 0.986 |  | |  | |  | 0.317 | 1.000 |  |  |
| 18-60 year | 187 (40.9) | 81 (27.8) |  |  |  | 76 (33.6) | 259 (30.7) |  |  |  | 34 (37.4) | 111 (38.4) |  |  |  | | 40 (40.8) | | 316 (35.2) |  |  |  |  |
| 61-85year | 270 (59.1) | 210 (72.2) |  |  |  | 150 (66.4) | 586 (69.3) |  |  |  | 57 (62.6) | 178 (61.6) |  |  |  | | 58 (59.2) | | 583 (64.8) |  |  |  |  |
| **Race, N (%)** |  |  | 0.025 | 0.999 |  |  |  | 0.745 | 0.942 |  |  |  | 0.907 | 0.993 |  | |  | |  | 0.505 | 1.000 |  |  |
| White | 377 (82.5) | 243 (83.5) |  |  |  | 190 (84.1) | 724 (85.7) |  |  |  | 78 (85.7) | 246 (85.1) |  |  |  | | 89 (90.8) | | 779 (86.7) |  |  |  |  |
| Black | 37 (8.10) | 34 (11.7) |  |  |  | 20 (8.85) | 62 (7.34) |  |  |  | 7 (7.69) | 26 (9.00) |  |  |  | | 4 (4.08) | | 55 (6.12) |  |  |  |  |
| Other | 43 (9.41) | 14 (4.81) |  |  |  | 16 (7.08) | 59 (6.98) |  |  |  | 6 (6.59) | 17 (5.88) |  |  |  | | 5 (5.10) | | 65 (7.23) |  |  |  |  |
| **Sex, N (%)** |  |  | <0.001 | 0.923 |  |  |  | 0.011 | 0.836 |  |  |  | 0.926 | 0.870 |  | |  | |  | 0.017 | 0.989 |  |  |
| Female | 109 (23.9) | 106 (36.4) |  |  |  | 48 (21.2) | 254 (30.1) |  |  |  | 36 (39.6) | 118 (40.8) |  |  |  | | 43(43.9) | | 282 (31.4) |  |  |  |  |
| Male | 348 (76.1) | 185 (63.6) |  |  |  | 178 (78.8) | 591 (69.9) |  |  |  | 55 (60.4) | 171 (59.2) |  |  |  | | 55 (56.1) | | 617 (68.6) |  |  |  |  |
| **Median household income, N (%)** |  |  | 0.001 | 0.812 |  |  |  | 0.173 | 0.990 |  |  |  | 0.674 | 0.949 |  | |  | |  | 0.031 | 0.962 |  |  |
| Less than $59999 | 139 (30.4) | 125 (43.0) |  |  |  | 78 (34.5) | 336 (39.8) |  |  |  | 33 (36.3) | 114 (39.4) |  |  |  | | 27 (27.6) | | 353 (39.3) |  |  |  |  |
| More than $60,000 | 318 (69.6) | 166 (57.0) |  |  |  | 148 (65.5) | 509 (60.2) |  |  |  | 58 (63.7) | 175 (60.6) |  |  |  | | 71 (72.4) | | 546 (60.7) |  |  |  |  |
| **With prior other cancer, N (%)** |  |  | 1.000 | 0.711 |  |  |  | 0.004 | 0.584 |  |  |  | 0.689 | 0.886 |  | |  | |  | 0.257 | 0.985 |  |  |
| No | 362 (79.2) | 230 (79.0) |  |  |  | 164 (72.6) | 689 (81.5) |  |  |  | 73 (80.2) | 224 (77.5) |  |  |  | | 77 (78.6) | | 752 (83.6) |  |  |  |  |
| Yes | 95 (20.8) | 61 (21.0) |  |  |  | 62 (27.4) | 156 (18.5) |  |  |  | 18 (19.8) | 65 (22.5) |  |  |  | | 21 (21.4) | | 147 (16.4) |  |  |  |  |
| **Laterality, N (%)** |  |  | 0.907 | 0.876 |  |  |  | 0.932 | 0.816 |  |  |  | 0.308 | 0.873 |  | |  | |  | 1.000 | 0.920 |  |  |
| Left | 231 (50.5) | 145 (49.8) |  |  |  | 119 (52.7) | 450 (53.3) |  |  |  | 41 (45.1) | 150 (51.9) |  |  |  | | 47 (48.0) | | 436 (48.5) |  |  |  |  |
| Right | 226 (49.5) | 146 (50.2) |  |  |  | 107 (47.3) | 395 (46.7) |  |  |  | 50 (54.9) | 139 (48.1) |  |  |  | | 51 (52.0) | | 463 (51.5) |  |  |  |  |
| **Size, median [IQR]** | 2.90 [2.20;3.50] | 3.30 [2.60;3.80] | <0.001 | 0.639 |  | 5.00 [4.50;5.80] | 5.60 [5.00;6.50] | <0.001 | 0.506 |  | 3.10 [2.50;3.50] | 3.50 [2.90;3.80] | 0.017 | 0.684 |  | | 5.20 [4.60;5.80] | | 5.70 [5.00;6.50] | <0.001 | 0.993 |  |  |
| **Histological type, N (%)** |  |  | 0.043 | 0.925 |  |  |  | <0.001 | 0.740 |  |  |  | 0.862 | 0.983 |  | |  | |  | 0.664 | 0.895 |  |  |
| Clear cell RCC | 247 (54.0) | 180 (61.9) |  |  |  | 129 (57.1) | 591 (69.9) |  |  |  | 67 (73.6) | 208 (72.0) |  |  |  | | 80 (81.6) | | 712 (79.2) |  |  |  |  |
| Other/undefined | 210 (46.0) | 111 (38.1) |  |  |  | 97 (42.9) | 254 (30.1) |  |  |  | 24 (26.4) | 81 (28.0) |  |  |  | | 18 (18.4) | | 187 (20.8) |  |  |  |  |
| **Sarcomatoid dedifferentiation, N (%)** |  |  | 1.000 | 0.915 |  |  |  | 0.091 | 0.881 |  |  |  | 0.317 | 0.973 |  | |  | |  | 1.000 | 0.904 |  |  |
| No | 448 (98.0) | 286 (98.3) |  |  |  | 218 (96.5) | 787 (93.1) |  |  |  | 90 (98.9) | 277 (95.8) |  |  |  | | 93 (94.9) | | 854 (95.0) |  |  |  |  |
| Yes | 9 (1.97) | 5 (1.72) |  |  |  | 8 (3.54) | 58 (6.86) |  |  |  | 1 (1.10) | 12 (4.15) |  |  |  | | 5 (5.10) | | 45 (5.01) |  |  |  |  |
| **Furhman grade, N (%)** |  |  | 0.272 | 0.969 |  |  |  | 0.019 | 0.830 |  |  |  | 1.000 | 0.791 |  | |  | |  | 0.303 | 0.907 |  |  |
| I/II | 302 (66.1) | 180 (61.9) |  |  |  | 125 (55.3) | 391 (46.3) |  |  |  | 51 (56.0) | 162 (56.1) |  |  |  | | 36 (36.7) | | 384 (42.7) |  |  |  |  |
| III/IV | 155 (33.9) | 111 (38.1) |  |  |  | 101 (44.7) | 454 (53.7) |  |  |  | 40 (44.0) | 127 (43.9) |  |  |  | | 62 (63.3) | | 515 (57.3) |  |  |  |  |
| **Regional lymph nodes removed, N (%)** |  |  | <0.001 | 0.896 |  |  |  | 0.038 | 0.999 |  |  |  | 0.014 | 0.984 |  | |  | |  | 0.017 | 0.970 |  |  |
| No | 446 (97.6) | 262 (90.0) |  |  |  | 211 (93.4) | 746 (88.3) |  |  |  | 88 (96.7) | 251 (86.9) |  |  |  | | 91 (92.9) | | 746 (83.0) |  |  |  |  |
| Yes | 11 (2.41) | 29 (9.97) |  |  |  | 15 (6.64) | 99 (11.7) |  |  |  | 3 (3.30) | 38 (13.1) |  |  |  | | 7 (7.14) | | 153 (17.0) |  |  |  |  |
| **Median time to event/censor, median [IQR]** | 4.17 [2.00;6.08] | 4.17 [2.12;6.17] | 0.958 | 0.575 |  | 3.50 [2.25;5.42] | 3.83 [2.08;6.17] | 0.158 | 0.646 |  | 3.33 [1.67;5.17] | 3.00 [1.92;5.17] | 0.458 | 0.454 |  | | 3.92 [2.02;4.92] | | 2.92 [1.75;4.83] | 0.068 | 0.026 |  |  |
| **Estimated median follow-up time, [95CI%]** | 4.58 [4.17-4.92] | 5.17 [4.58-5.67] | 0.052 | 0.052 |  | 4.25 [3.83-4.75] | 5.00 [4.67-5.33] | <0.001 | <0.001 |  | 3.42 [2.50-4.33] | 3.58 [3.08-4.17] | 0.110 | 0.110 |  | | 4.17 [3.58-4.67] | | 3.50 [3.33-3.75] | 0.740 | 0.740 |  |  |
| **Survival outcomes, N (%)** |  |  | <0.001 | 0.011 |  |  |  | 0.035 | 0.475 |  |  |  | 0.244 | 0.623 |  | |  | |  | 0.706 | 0.675 |  |  |
| Alive | 410 (89.7) | 226 (77.7) |  |  |  | 184 (81.4) | 618 (73.1) |  |  |  | 83 (91.2) | 243 (84.1) |  |  |  | | 83 (84.7) | | 736 (81.9) |  |  |  |  |
| Dead from RCC | 8 (1.75) | 21 (7.22) |  |  |  | 16 (7.08) | 96 (11.4) |  |  |  | 2 (2.20) | 16 (5.54) |  |  |  | | 8 (8.16) | | 76 (8.45) |  |  |  |  |
| Dead from other events | 39 (8.53) | 44 (15.1) |  |  |  | 26 (11.5) | 131 (15.5) |  |  |  | 6 (6.59) | 30 (10.4) |  |  |  | | 7 (7.14) | | 87 (9.68) |  |  |  |  |
| RCC: renal cell carcinoma; PN: Partial nephrectomy; RN: Radical nephrectomy; OR: odds ratio; IQR: interquartile range; CI: confidence interval; FI: perirenal fat invasion; SI: sinus/perisinus fat invasion; | | | | | | | | | | | | | | | | | | | | | | | |
